# UK medical education on human trafficking: assessing uptake of the opportunity to shape awareness, safeguarding and referral in the curriculum

**DOI:** 10.1186/s12909-018-1226-y

**Published:** 2018-06-13

**Authors:** Poojani Arulrajah, Sarah Steele

**Affiliations:** 1 Barts and The London School of Medicine and Dentistry, London, UK; 2 Jesus College, Cambridge and the University of Cambridge, Cambridge, UK

**Keywords:** Human trafficking, Medical education, United Kingdom, Safeguarding, Training

## Abstract

**Background:**

Human trafficking is a serious violation of human rights, with numerous consequences for health and wellbeing. Recent law and policy reforms mean that clinicians now hold a crucial role in national strategies. 2015 research, however, indicates a serious shortfall in knowledge and confidence among healthcare professionals in the UK, leading potentially to failures in safeguarding and appropriate referral. Medical education is a central point for trafficking training. We ascertain the extent of such training in UK Medical Schools, and current curricular design.

**Method:**

We sent Freedom of Information requests to the 34 public UK medical schools, which included a preliminary question on education provision, supplemented with follow-up questions exploring the nature, delivery and format of any education, as well as future curriculum development.

**Results:**

There was a response rate of 97%. A majority (72%) of the schools did not provide trafficking education. 13% of these did, however, offer opportunities outside the formal curriculum. 70% had no plans to implement any education opportunities. Among the 28% of schools providing teaching, 56% integrated this within the core curriculum. 56% only delivered this within a single year of the degree. 67% provided some form of teaching in-person, while 78% used a combination of methods.

**Conclusion:**

Medical education on trafficking in the UK is variable and often absent. To produce future clinicians who are competent and capable, there is a need for expanded education on trafficking and research into optimal curriculum design. The UK’s new Independent Anti-Slavery Commissioner should work with medical schools to develop an educational strategy urgently to fulfil the UK Government’s plans and commitments. Both in the UK and around the world, human trafficking education presents a critical opportunity to address human rights and safeguarding to a generation of new doctors.

## Background

For over two decades now, trafficking in persons—also referred to by the broader moniker of modern slavery—has attracted extensive international attention. A reprehensible violation of human rights, this crime involves the recruitment and movement of individuals, both domestically and internationally, into conditions of exploitation [1]. Estimates suggest more than 2.4 million people are trafficked worldwide, with the refugee crisis expected to further drive the trade as migrants make risky choices in trying to find a better life [2]. Those subjected to trafficking are exploited across the sexual trade (which includes prostitution, escort work and pornography), in forced labour (occurring usually in the manufacturing, farming, construction, entertainment and travel industries), through organ harvesting, and in domestic servitude, amongst other areas [3, 4]. In the United Kingdom (UK), it is estimated that between 10 and 13,000 people are believed to be exploited annually, however the Government acknowledged when passing the Modern Slavery Act 2015 that far fewer are actually aided due to failings across the UK strategy [5].

Centrally, the new Act stresses the need for public authorities to better cooperate in the assistance of trafficked people, identification of victims, and provision of support [5]. The new Independent Anti-Slavery Commissioner appointed under the Act placed the National Health Service (NHS) at the heart of his new approach [6]. The Commissioner’s Strategic Plan for 2015–17 expanded on the safeguarding requirements of healthcare professionals — those measures put into place to protect both adults and children’s rights to live in safety, free from abuse and neglect — calling for improved identification and effective response to victims of modern slavery through the promotion of awareness-raising and training of healthcare professionals across the UK, and collaboration with the NHS to ensure it is delivered [6]. This is supported by the General Medical Council’s (GMC) declaration that it a priority for clinicians to undertake safeguarding duties in protecting the rights and welfare of vulnerable adults and children in their care [7]. Therefore, at present, the UK’s Human Trafficking Strategy requires the NHS to work with partner organisations to respond to human trafficking through safeguarding and protecting vulnerable adults and children at risk of exploitation, thus making their safety a shared endeavour. At the same time, the Government encourages NHS professionals to voluntarily report any suspected incidents using new anonymous reporting methods, facilitating the better collection of data to direct funding and resources [6].

However, a 2015 study conducted by Ross and colleagues found that 87% of NHS professionals reported a lack of knowledge in identifying trafficked people, and 78% reported insufficient training limiting their ability to assist these individuals [8]. Similar trends are observed across the world [9, 10]. However, this gap in knowledge is not simply a matter of the phenomenon being newly conceived and training yet to have flowed across to clinicians. Importantly, existing research confirms educational intervention increased health professionals’ knowledge and recognition of trafficked individuals, so the gap evidences a lack of training across qualification and practice [11]. Troublingly, medical students around the world also exhibit the knowledge gap. In Canada, for example, research done into medical students’ awareness and attitudes to human trafficking found that 94% reported having little to no knowledge of the subject, and amongst those who did, most had first learnt of it prior to medical school [12].

Such a lack of awareness and training are not, however, the consequence of low prioritisation or perceived irrelevance. Existing research indicates that clinicians would welcome training on human trafficking, specifically on how to identify and respond to human trafficking [8, 11]. Students also feel the topic should be taught in medical schools [12]. Crucially, in discussing the training desired, UK healthcare workers reflected that while they generally knew what trafficking is, they needed better training on how to talk to patients about experiences of human trafficking, and how to make referrals to local and national support services [8]. Such findings are troubling as inappropriate questioning of the patient, or non-consensual or incorrect referral, may both have significant impacts on the patient’s safety and long term well-being [5, 6]. It is critical for training demands to be met with adequate education and information provision.

Notably, in the UK, despite such requests for training, medical schools hold no legal obligation to provide such training. However, the General Medical Council’s (GMC) standards for medical education insure that certain fundamentals of safeguarding are incorporated into the curriculum [13], with *Tomorrow’s Doctors* requiring medical schools to provide the appropriate education and training that enables its students to ‘[i]dentify the signs that suggest children or other vulnerable people may be suffering from abuse or neglect and know what action to take to safeguard their welfare’ [14].^p.21^ Alongside the *International Framework for Action To Implement the Trafficking in Persons Protocol 2009*—a technical assistance tool published by the United Nations that promotes the inclusion of training modules on human trafficking in medical, psychological and social service curricula—such safeguarding training requirements make it critical that medical students, who will be placed within the clinical setting both during their degrees and beyond, understand trafficking and appropriate responses [15]. Indeed, as the GMC confirms, the training of clinicians—key decision makers within a multi-disciplinary team—is a lifelong process, the foundations of which occur in the undergraduate years [14].

We seek to ascertain the extent to which medical students are afforded the opportunity to learn of human trafficking before entering the NHS through training in the formal curricular. To date, there has currently been no research on the education on human trafficking offered to medical students in universities in the UK that the authors are aware of. We therefore surveyed medical schools to assess the proportion of medical schools providing education on trafficking, while identifying the context and format of any education provided [16]. Such research enables us to explore any education gap, as well as whether there is uniformity and consistency in education provision. We explore opportunities to improve educational offerings in order to produce future clinicians who deliver safe and excellent care to trafficked persons, while fulfilling national and international obligations.

## Methods

### Study design

We used a Freedom of Information (FOI) request method to collect data [16]. The Freedom of Information Act 2000 and the Freedom of Information Act (Scotland) Act 2002 (hereafter ‘FOI Acts’) are laws facilitating the right to access information held by public sector organisations, including universities receiving public funding, in England, Scotland, Wales and Northern Ireland [16, 17]. Requests made under the Acts oblige public bodies to disclose recorded information about their activities within the statutory time period of 20 working days. The requests are subject to certain exemptions, including confidentiality and cost-restraints [16]. Such exemptions make it important to craft a request that ensures the appropriate and maximum amount of information is collected, while complying with regulations, and that the body is one governed by the Acts in the first instance.

The request comprised of a preliminary closed question, followed by a series of follow up questions that corresponded to a “yes” or “no” response to the initial question. The first question asked “Do you provide training or any teaching to medical students on the subject of human trafficking, either exclusively or within safeguarding training?”. If the answer was “YES”, respondents were asked:

- Within what context is the teaching/training delivered? – is it integrated in to the core curriculum (whereby *all* medical students have to learn about it), or is it a voluntary/elective module (whereby *those who choose* to study the subject may do so)?
- In which year of the medical (MBBS) programme is the teaching/training delivered or available to be electively selected? - Year 1,2,3,4, or 5?
- How is the training or teaching delivered? – Does it involve or take the form of lectures, clinical teaching, videos, online, face-to-face tutorials or group/seminar work?

If “NO”, respondents were asked if there were plans being developed to provide training or teaching for medical students on the subject of human trafficking (either within safeguarding or beyond). A definition of human trafficking was provided to reduce confusion and respondents were advised to contact the researcher in the event clarification was needed.

### Study population

**Fig. 1:**
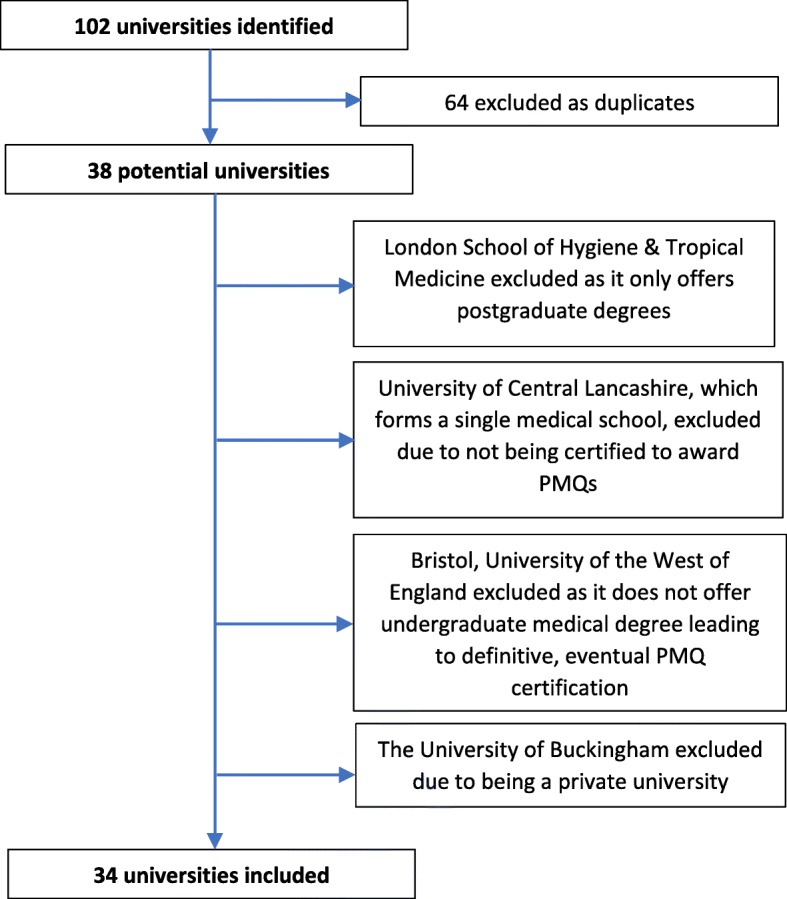
Flow Diagram of Selecting the Sample of Medical Schools

We devised a list of current medical schools using the General Medical Council’s publically available list of medical schools [18], cross-checked this with the Medical Schools Council website and Universities and Colleges Admissions Service (UCAS) [19, 20]. The use of three databases was necessary as pathways through medical education in the UK are variable, and some databases only listed institutions offering primary medical qualifications (PMQ), while others included divided programmes, where students take degrees across institutions moving to be awarded the PMQ after initial study at another institution. The overall learning experience of students, which shapes them as clinicians, takes place across both institutions, and thus both needed to be included in the sample. Also, the only private university- the University of Buckingham- was excluded from the sample due to it not being governed by the FOI provisions. Figure 1 shows the selection process, and the list of medical schools is provided as a supplemental document.

### Collection and analysis

The request (available as a supplemental document) was sent to these 34 universities using email correspondence. A preliminary closed question on provision of human trafficking education was supplemented with a set of follow-up questions, which facilitated the calculation of quantitative data in terms of proportionality, and qualitative data to assess context and form of education. Responses were recorded in Excel for analysis.

### Research ethics

While FOIs request data held by public bodies to foster both openness and accountability, we sough ethics approval from Queen Mary, University of London, which confirmed that a study of this nature is exempt. Also, while the data are publically available, it was decided that they should be handled entirely anonymously so that no individual school is open to criticism. All data were de-identified.

## Results

Reponses were received from thirty-three medical schools; a 97% response rate. Among those who responded, one university replied that it did not hold any recorded information relevant to the request, and thus was excluded from the analysis. Therefore, only thirty-two responses were analysed.

Of these schools, nine (28%) indicated they provide teaching to medical students on trafficking. Three of these schools (33%) were categorised as ‘yes BUT limited’ due to reservations noted in their responses, including phrases such as “may arise” or “limited information”. For example, one medical school located in the South of England mentioned that its adult safeguarding training makes reference to human trafficking, but “not in any detail”.

Of the responses received, twenty-three schools (72%) indicated they are not providing any teaching on human trafficking. Among them, three (13%) noted opportunities outside the formal medical curriculum. For example, a Scottish university mentioned its intercalated BSc programme, called ‘Global Health in a Primary Care Context’, which covers human trafficking and is open for medical students through a year out from the medical programme.

### Context and format of education

Of the responses analysed, the nine that indicated they provide education were further analysed to identify the context and format of that education. Of these, five schools indicated that the teaching or training was integrated into the core curriculum, making it compulsory for all medical students to learn about trafficking. Only one medical school in the Midlands offered a voluntary safeguarding training workshop that discusses human trafficking. Notably, three medical schools offered more than one programme or module that teaches about human trafficking. For example, a medical school in the East of England offers human trafficking teaching through an elective class, called ‘Global Health’, within the Students Allied to Medicine module, and a compulsory adult safeguarding class.

Specifically, five medical schools (56%) indicated that education was only offered in one year of the typically five-year degree. Another two medical schools (22%) indicated that two years of their medical programmes contained human trafficking education, and the remaining two medical schools indicated providing more than two years of education. Among them was a medical school in the East of England that offered a mixture of core and elective modules in the 2nd, 3rd and 4th years of their medical programme, and a school in Northern Ireland that offered the highest number of total years, with the 1st, 3rd, 4th and 5th years of the medical programme containing human trafficking education. Three medical schools (60%), which had only one year of the medical programme containing human trafficking education, offered it during the 3rd year, and two (40%) offered it during the 5th year.

We also identified that five medical schools (56%) provided teaching in person, while four medical schools (44%) provided teaching both through self-directed learning and in person, and none of them used only self-directed learning. With regard to the style of teaching, seven medical schools (78%) used a combination of methods to deliver education on trafficking. In person methods included, lectures (*n* = 8), seminars and workshops (*n* = 6), case studies (*n* = 6), videos (*n* = 2), games (*n* = 1) and shadowing professionals in the health and social sector (*n* = 1). E-learning resources, used by three medical schools (33%), were the most popular teaching method used in self-directed learning, with a medical school in the East of England utilising the same online toolkit created by the Department of Health [21]. The other self-directed method employed was independent reading (*n* = 1).

### Future plans

Among the 23 schools not currently providing education, only 22 responded about future plans, with a London medical school and a Scottish medical school replying that they did not hold any recorded information on this question. We therefore only analysed 20 responses. Of these, 14 (70%) indicated not having any plans to provide education on human trafficking, while five (25%) indicated having possible future plans by mentioning the medical curriculum was under review and development. A medical school in Scotland (5%) indicated that it was currently in the process of introducing the topic into the curriculum.

## Discussion

The purpose of this article was to ascertain the provision, context, and delivery of, human trafficking education in the current curriculum for medical students in the UK. The high response rate to our survey increases the validity, reliability, and generalisability, of the data. As such, it provides a good indication of the current status of the medical curriculum pertaining to human trafficking in the UK, and by extension an indication of the early factors underpinning the lack of knowledge on the topic observed in professional practice.

### Limitations

However, this study had a number of limitations. By focusing solely on medical schools in the UK, the study provides a geographically limited understanding of why gaps in knowledge occur amongst NHS clinicians. Studies indicate as many as 26% of NHS doctors are overseas qualified, and as such are likely to have attended non-UK based medical schools [22]. Other studies both of overseas qualifications and the curricular for UK conversion are therefore desirable.

Also, by using an FOI method there was the possibility that the questions were open to interpretation on the respondents’ part, and thus careful phrasing was essential. A number of measures were taken to minimise interpretation, including providing explicit definitions on “human trafficking”, while also encouraging the university to contact the requester if clarification was required. Where ambiguous responses were received, clarification was sought from the medical school.

Furthermore, this study did not assess the content of the education in terms of its relevance to clinical practice, the duration of the training, and if, and what, types of assessments were involved. Future studies should build on this article to crosscheck the curriculum against the National Referral Mechanism indicators, or the toolkit published by the Department of Health, to provide a more complete understanding of this topic [21, 23].

### Next steps

Troublingly, we found that 72% of public medical schools in the UK do not provide any trafficking education. This may be the result of legal obligations being placed on the NHS to deliver training on safeguarding, leading medical schools to conclude training will take place in the Foundation Years. Alternatively, this gap may be due to the fact that safeguarding objectives and requirements set by the GMC on medical schools do not explicitly mention human trafficking. Understanding why 70% of medical schools not offering training have no plans to implement human trafficking education in the future is therefore critical, as such a lack of training may be feeding into the lack of knowledge and confidence in NHS doctors recently observed by Ross and colleagues [8]. This may be a point of enquiry for the Independent Anti-Slavery Commissioner in improving training across the UK.

In the minority of medical schools providing trafficking education (*n* = 9), some indicated it was delivered solely through safeguarding training, and that it was part of broader discussion. Such statements indicate training may be inadequate or piecemeal. Certainly, comments revealed the use of safeguarding as an umbrella-term encompassing an array of abuse and neglect, with one university directly identify its package was aimed at identifying sexual abuse and vulnerability, but mentioned that “[t]here is not a specific learning objective about human trafficking”. Such a response indicates the over-simplification of trafficking to sex trafficking in the minds of educators, which should be explored, as this is out of alignment with UK law. It may also leave students with an incomplete understanding of non-sexual forms of exploitation and drive inadequate or unsafe responses in later clinical practice, as the methods for identification and initial contact, the referral procedures, and the guidelines surrounding informing law enforcement agencies can vastly differ between different forms of abuse [24, 25]. Training of clinical educators by researchers and Third Sector organisations may improve such shortfalls.

Critically, our results also indicate a student demand for education on the issue. Three institutions provide opportunities outside the formal curriculum to learn about human trafficking, evidencing that medical students are keen to learn about the subject and are taking up options to obtain the relevant education when it is not in the core curriculum. These results confirm those identified in the Canadian study which identified that 85% of students felt it was important or very important to learn about the identification and healthcare needs of trafficked individuals [12]. In light of such findings, building learning opportunities into the formal curriculum should drive student interest and engagement, a point that should be made to the institutions indicating no plans to implement any teaching on the issue.

However, curriculum development should be undertaken in light of greater research and findings on good practice, both in the UK and abroad. We identified great variations in the context and format of teaching when it was delivered, which affirms general research on medical education [26]. It is therefore important to research which methods and content are most effective in improving clinician responses to this form of abuse. Notably, studies indicate that even a short, single-session of education on human trafficking significantly increased the knowledge and self-reported recognition of trafficked individuals among healthcare professionals [11], which indicates even brief incorporation may improve the issues identified in the NHS by Ross and colleagues [8]. Critically, the Independent Anti-Slavery Commissioner is positioned to provide information, education or training to encourage good practice in the identification of trafficked individuals, and thus should hold a central role in developing curricula across the UK medical schools [5, 6]. Distinctions should be made for undergraduate and postgraduate level training to account for the differing needs of students and professionals. This will help medical schools adopt evidence-based techniques that are both effective and consistent across the UK in delivering human trafficking education.

However, just as modern slavery is not an issue confined to one country, its responses require international cooperation and cross-national learning. It is essential that innovations in teaching and learning are shared both within countries and between countries, as school-based and national curriculum developments may inform better educational practice around the world. Responses to encourage collaboration and cross-learning globally, and to facilitate the creation of broader and more effective approaches in the educational and clinical settings, must be implemented.

## Conclusions

In sum, medical students, as future clinicians, have a crucial and strategic need for education that allows them to perform a critical role in intervening, identifying, assessing and referring trafficked individuals. The safety and quality of care delivered to trafficked persons requires clinicians to have a good knowledge both of trafficking, including clinical indicators, and appropriate responses both within the clinic and to the relevant referral bodies. Despite a renewed focus on this subject in recent decades, there are serious shortcomings within the health sector in meeting and addressing the needs of modern slaves, leading to behaviours that can jeopardise the well-being of these already vulnerable adults and children. We recommend expanding education on modern slavery within UK medical schools to establish the foundation for best practice in future clinicians.

## Abbreviations

BSc: Bachelor of Sciences
FOI: Freedom of Information Act request
GMC: General Medical Council
MBBS: Medicinae Baccalaureus, also known as Bachelor of Medicine, Bachelor of Surgery
NHS: National Health Service
PMQ: Primary Medical Qualification
UCAS: Universities and Colleges Admissions Service
UK: United Kingdom
